# Translating GWAS in rheumatic disease: approaches to establishing mechanism and function for genetic associations with ankylosing spondylitis

**DOI:** 10.1093/bfgp/ely015

**Published:** 2018-05-05

**Authors:** Julie A Osgood, Julian C Knight

**Affiliations:** 1 Functional genomics of ankylosing spondylitis, University of Oxford, Oxford, UK; 2 Wellcome Centre for Human Genetics, University of Oxford, Oxford, UK

**Keywords:** ankylosing spondylitis, genome-wide association study, regulatory genetic variants, expression quantitative trait loci, chromatin conformation capture

## Abstract

Ankylosing spondylitis (AS) is a highly heritable chronic inflammatory arthritis characterized by osteoproliferation, fusion of affected joints and systemic manifestations. Many disease associations for AS have been reported through genome-wide association studies; however, identifying modulated genes and functional mechanism remains challenging. This review summarizes current genetic associations involving AS and describes strategic approaches for functional follow-up of disease-associated variants. Fine mapping using methods leveraging Bayesian approaches are outlined. Evidence highlighting the importance of context specificity for regulatory variants is reviewed, noting current evidence in AS for the relevant cell and tissue type to conduct such analyses. Technological advances for understanding the regulatory landscape within which functional variants may act are discussed using exemplars. Approaches include defining regulatory elements based on chromatin accessibility, effects of variants on genes at a distance through evidence of physical interactions (chromatin conformation capture), expression quantitative trait loci mapping and single-cell methodologies. Opportunities for mechanistic studies to investigate the function of specific variants, regulatory elements and genes enabled by genome editing using clustered regularly interspaced short palindromic repeats/Cas9 are also described. Further progress in our understanding of the genetics of AS through functional genomic and epigenomic approaches offers new opportunities to understand mechanism and develop innovative treatments.

## Introduction

**Figure 1:**
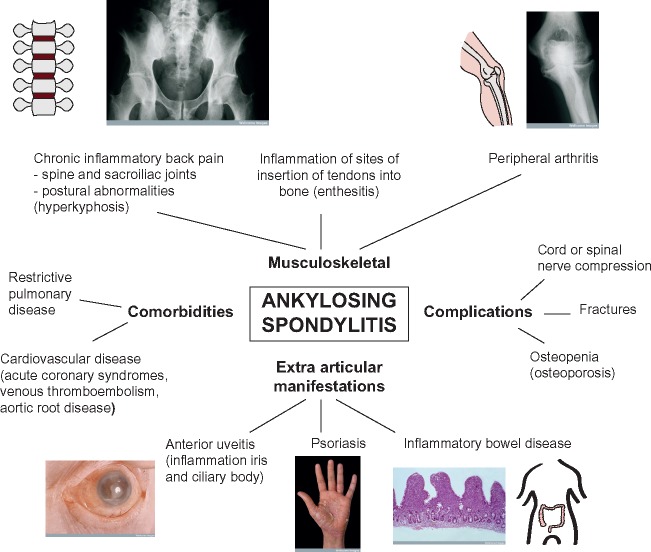
Overview of the musculoskeletal and extra-articular manifestations of AS together with comorbidities and major complications. Images from Wellcome Images under Creative Commons.

**Figure 2:**
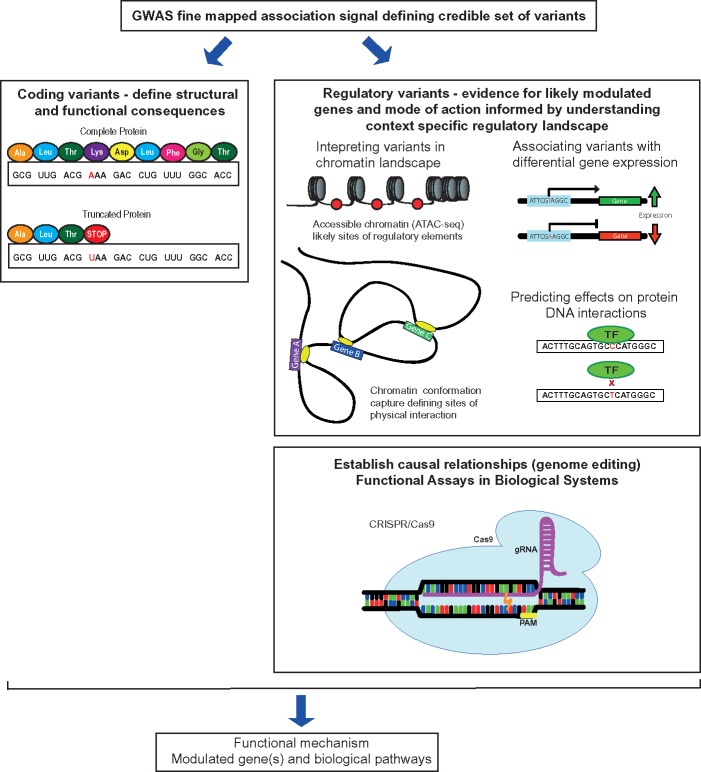
Illustration of some approaches enabling understanding of the functional basis of GWAS.

Ankylosing spondylitis (AS) is a severe chronic inflammatory arthritis and part of a group of diseases collectively known as spondyloarthropathies [1]. AS is characterized by inflammation and osteoproliferation, followed by bone fusion of affected areas, which are typically the spine and sacroiliac joints (Figure 1). The disease affects men two to three times more often than women and shares many features seen in other immune-mediated traits, including peripheral arthritis, anterior uveitis, psoriasis, inflammatory bowel disease (IBD), osteoporosis and cardiovascular disease (aortitis, aortic valve disease, conduction disturbances, cardiomyopathy and ischaemic heart disease) [2]. It is associated with significant morbidity and mortality, including chronic pain, disability and accompanying comorbidities such as cardiovascular and gastrointestinal complications. This has a significant socio-economic impact, as AS particularly affects young adults [3]. The introduction of biologic therapies [4] highlights significant opportunities for improved care but currently do not affect disease progression, and the pathophysiology of AS remains relatively poorly understood [5, 6], limiting early effective intervention. Genetics offers significant opportunities to address this challenge given the high heritability of AS [7, 8]. Following the early discoveries in the 1970s regarding the role of human leukocyte antigen (HLA), substantial recent progress has been made in establishing genetic susceptibility to AS through genome-wide association studies (GWASs) [9–12]. This has the potential to provide new insights into disease pathogenesis and opportunities for therapeutic intervention. However, our current ability to establish function and mechanism for reported genetic associations is limited, requiring new approaches and innovative strategies to maximize the translational impact of GWAS in AS. In this article, we outline current progress and future strategies for addressing this important area of unmet need (Figure 2).

## Genetics of AS

AS is a highly heritable disease with first-degree relatives being >50 times more likely to develop the disease than unrelated individuals, a parent–child recurrence risk of 7.9% and overall heritability of 90% [7, 8, 13]. An early breakthrough in genetic research into AS came in 1973 with the landmark discovery of association between disease occurrence and the major histocompatibility complex (MHC) Class 1 allele, HLA-B27 [14, 15]. Among AS patients, 96% are HLA-B27 positive however, only a minority of HLA-B27 positive individuals develop AS, and current understanding suggests that while HLA-B27 is the strongest genetic risk factor it is not sufficient for the disease to develop [7, 13, 16–18]. Monozygotic twins have a disease concordance rate of 63%, compared with 12.5% for dizygotic twins (27% in HLA-B27 positive dizygotic twins) [7].

**Figure 3:**
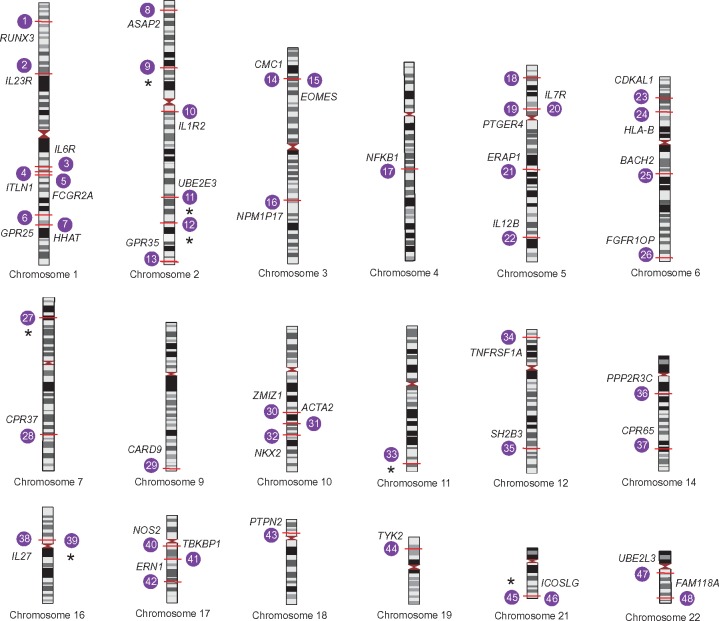
Overview of AS GWAS loci. Loci associated with AS at genome-wide significance reported by Ellinghaus *et al.* [20] are plotted (numbered 1–48). The implicated gene and chromosomal position (marked in red) are shown for each locus; where there is no reported gene, this is indicated by an astrerix. In the majority of cases, the genes listed are candidates based on proximity and biological plausibility, and causality has not been established except in a small number of instances.

The first genome-wide association study for AS was performed by the Wellcome Trust Case Consortium and the Australo-Anglo-American Spondylitis Consortium in 2007 [17]. This focused on a genome-wide set of 14 436 non-synonymous SNPs together with 897 MHC SNPs, identifying *ERAP1* and *IL23R* as new susceptibility loci. Subsequent GWAS and Immunochip studies identified 48 genomic loci reaching genome-wide significance and have further highlighted the role of non-MHC genes (Figure 3) [9, 11, 12, 17, 18]. Most disease-associated GWAS SNPs are not found in exons but rather in intronic and intergenic regions with putative regulatory effects [19, 20] and those associated with AS are no exception, including, for example, associations in at least two gene deserts. Many such regulatory variants can be key modulators of gene expression through generation or disruption of splice sites, change in transcription factor affinity, altered physical chromatin interactions, change in microRNA action or modification of DNA methylation, resulting in increased or decreased chromatin accessibility [21–24]. All of these can cause local effects, or can act at a distance, meaning the modulated gene(s) responsible for the observed disease association with the genetic marker can be hard to establish. Some of the most notable genetic findings from recent studies involving AS are the discoveries implicating involvement of the aminopeptidases, ERAP1 and ERAP2, and genes in both the tumor necrosis factor (TNF) and Interleukin-23 (IL-23) pathways [9, 11, 17].

*ERAP1* is a promising candidate for disease, and is an important example of epistasis, as it has been shown to be associated with AS only in HLA-B27 positive individuals [11]. *ERAP1* encodes an aminopeptidase involved in the processing of peptides before their presentation on MHC Class I molecules [25, 26]. There is evidence that an AS-protective single nucleotide polymorphism (SNP), rs31087, has a decreased ability to cleave peptides before MHC presentation and therefore slows down the rate of cleavage [11, 27], but the associations are complex with several different alleles involved [28]. A decrease in ERAP1 activity has also been associated with a decrease in the stability of HLA-B27 [29]. Chen and colleagues [30] examined the effect of silencing ERAP and found that there was an extension of both the N and C terminals of HLA-B27 epitopes in the absence of ERAP. It has also been found that ERAP1 activity governs HLA-B27 free heavy chain expression on the cell surface and may even be involved in Th17 promotion [31]. However, the exact mechanisms on how these findings may relate to disease remain unclear and more experimental evidence is required. ERAP2 has also been implicated in disease [9, 17]. It is a similar aminopeptidase responsible for N terminal cleavage of peptides before antigen presentation, and can form a heterodimer with ERAP1 [32, 33]. Other aminopeptidase genes associated with AS are *LNPEP* and *NPEPPS* [9].

IL-23 is a major proinflammatory cytokine, and several genes involved in IL-23 signalling have been implicated in AS through genetic studies, including tyrosine kinase 2 (*TYK2*), *IL12*, nuclear factor kappa B subunit 1 (*NFKB1*) and *IL23R*, encoding the receptor for IL-23 [9, 17, 18, 34]. SNPs found in *IL23R* have also been shown to be associated with other autoimmune diseases such as psoriasis [35], Crohn's disease [36], IBD [37], ulcerative colitis [38], psoriatic arthritis [39] and Behcet's disease [40]. On ligation with IL-23, IL23R initiates a signalling cascade through JAK2 and STAT3, leading to the production of IL-17 and IL-22, all of which are involved in inflammation. IL-22 is also thought be implicated with the expression of genes involved in new bone growth [41].

GWASs have also uncovered SNPs near/in three major genes in the TNF pathway: *TNFRSF1A* [11, 17, 42–44], *TRADD* [45] and *TNFSF15* [46]. TNF is an important mediator of inflammation and remains the main target for biologic treatments [47]. It is proposed that TNF is involved in the initial inflammation of joints, which then leads to bone degradation followed by new bone growth and fusion of joints as a reparative attempt [48]. However, evidence from animal models suggests that anti-TNF therapies help treat inflammation but not the bone growth [49–51].

## Fine mapping and resolving likely causal variants from GWAS

A major challenge to the translation of GWAS into mechanistic understanding is determining the causal variant(s), as this may not have been directly genotyped, and typically, there are many SNPs in linkage disequilibrium with the lead GWAS marker(s). Fine-mapping approaches often use a combination of statistics and functional annotations for variants. These include the use of genotyping arrays developed to study a specific set of SNPs, such as the Immunochip for immune-relevant variants, and statistical approaches that can define a small subset of statically likely casual SNPs, known as a credible set, that can then undergo functional annotations through the use of data sets and online predictors. Fine mapping typically involves imputation [52] and targeted re-sequencing [53] with stepwise conditional analysis to define independent association signals within a locus [54]. To narrow down the associations to particular variants, calculating *a posterior* probability in a Bayesian approach can define a credible set [54]. Credible sets can then be subjected to functional annotations such as arising from ENCODE [55] and NIH Roadmap [56] studies. A Bayesian fine-mapping approach has been used to examine 50 susceptibility loci for type 1 diabetes [57]. The authors identified 29 SNPs in 99% credible sets for enhancer regions in the thymus, T cells, B cells and CD34+ stem cells, allowing for future functional assays to be carried out using a manageable set of potential causative variants. Application in AS is enabled by international collaborative efforts to maximize samples sizes and power for GWAS such as through the International Genetics of Ankylosing Spondylitis Consortium (IGAS).

## Characterizing role of causal genetic variants in landscape of gene regulation

Given the majority of GWAS SNPs for complex traits are found within introns and intergenic regions, considering the diversity of effects on gene regulation involving allelic variation is essential [19]. Moreover, knowing how the genotype of a non-coding variant relates to gene expression is complex. For example, a single non-coding SNP may modulate the regulation of multiple genes through a given enhancer and multiple SNPs may exert a combined (haplotypic) effect on the regulation of a given gene with evidence that SNPs in linkage disequilibrium (LD) can act on multiple enhancers, all near each other, and collectively contribute to observed expression of a gene [58]. Complex processes govern gene regulation including post-translational modifications, such as methylation and acetylation, the physical conformation and looping of chromatin to bring, for example, enhancer elements proximal to regulated genes, microRNA activity, relative DNA-binding affinity for transcription factors and alternative splicing [21, 22, 24, 59]. Some of these processes are context specific in that an enhancer may only be active in a certain cell type or under a particular stimulus, or that regions of open chromatin will vary between cell types [60–62]. Variants that impact transcription factor-binding motifs, chromatin accessibly or physical chromatin interactions could all have significant effects on gene expression and relate to disease. Therefore, to better understand the role of non-coding variants in disease pathogenesis, there is value in understanding the chromatin landscape across cell types and its interactions with both nearby and distant elements.

### Defining chromatin accessibility

A highly informative predictor of the location of regulatory elements is identification of regions of open chromatin accessible to transcription factors, co-regulatory factors and chromatin interactions. DNase I mapping has been the golden standard for defining accessible chromatin and was used by the ENCODE Consortium for genome wide mapping of open chromatin regions [55, 63]. It relies on the use of the DNase I enzyme to create double-stranded breaks in regions of accessible open chromatin to generate fragments [64]. These fragments are then amplified and analysed by Southern blotting, real-time quantitative polymerase chain reaction (PCR) or next-generation sequencing to provide a visual map of regions of open chromatin [65, 66]. The assay for transposase-accessible chromatin using high-throughput sequencing (ATAC-Seq) is a more recently developed method to assay chromatin accessibility and was first introduced in 2013 by Buenrostro *et al.* [67] as an improvement on other methods used to perform epigenetic profiling. Like DNase I mapping, it is able to evaluate transcription factor-binding sites, nucleosome positions and regions of open chromatin in a single assay. However, unlike DNaseI, it works effectively with low cell numbers, maps transcription factor and nucleosome binding, and is credited with being more robust [63, 67]. Fragmentation is achieved using a Tn5 transposase that has the ability to not only fragment the DNA but will also simultaneously tag it with sequencing adaptors. In regions where the DNA is tightly wound around histones or bound by proteins, the transposase will be unable to cut the DNA, and therefore data are generated about where chromatin is easily accessible for DNA-binding proteins. This type of data can be overlapped with GWAS hits to annotate which variants are likely to be involved in gene regulation, and which are non-functional SNPs in regions of dense chromatin. For example, ATAC-seq was used recently to help prioritize schizophrenia GWAS risk variants. Forrest *et al.* [68] used excitatory neuronal differentiation from hiPSCs to examine genome-wide areas of open chromatin using ATAC-seq. From this, they were able to prioritize potential risk variants affecting neural development and at a specific risk locus, *MIR137*, narrowed down the list of potential causal SNPs to just one found in open chromatin. Furthermore, they were able to confirm using CRISPR that this SNP decreased expression of *MIR137*.

Genetic variants can influence gene expression by disrupting transcription factor binding, creating or removing methylation sites, changing splice sites, altering nucleosome structure or create new promoter-like elements [69–72]. These changes in gene expression can be of small magnitude or substantial, as seen with loss of GATA-1 binding associated with a single-nucleotide change in the *DARC* promoter in Duffy blood group antigen-negative individuals [73]. Mutations that change methylation sites can cause genes to become hypo- or hyper-methylated and can result in significant changes in expression by creating or removing areas in which proteins can bind to initiate transcription [70]. The acetylation of histones can also have an impact on gene expression and disease. Rubinstein–Taybi syndrome is an autosomal dominant disorder characterized by developmental delay and congenital abnormalities. It has been associated with mutations in the cAMP response element-binding protein, a histone acetyl transferase, which inhibit its function and decreases transcription [74]. Modulation of splicing is a further important mechanism by which genetic variants may act, reported for example with *CTLA4* and associated with autoimmunity [75], while effects on mRNA stability may also be important as illustrated by *CDSN* and susceptibility to psoriasis [76]. Uncovering functional variants modulating such mechanisms would be a major step in understanding the underlying pathogenesis of AS.

### Defining chromatin interactions

Understanding the 3D landscape of the genome is another current challenge to characterizing non-coding disease variants. Distant, physical interactions between *cis*-acting regulatory elements and promoters can occur to regulate transcription through the bending and folding of chromatin. Topologically associated domains (TADs) describe regions of frequent physical chromatin interactions [77]. Cohesion and the transcriptional repressor CCCTC-binding factor help regulate TAD boarders and facilitate interactions within, including bringing enhancer elements close to different promoters [78]. Chromatin is highly likely to interact with another region of chromatin within the same TAD to influence the expression of a gene, but unlikely to interact with chromatin from another TAD [79, 80]. Therefore, understanding these 3D interactions provides insight into the more complex regulation of gene expression and helps further annotate GWAS SNPs. Chromatin conformation capture (3C) is one approach to analyse these physical interactions and provide evidence connecting regions of the genome with the promoters they interact with, and can therefore associate SNPs with the genes they may regulate [81]. In 3C, DNA is crosslinked within the cell and then digested and re-ligated. Chromatin that physically interacts will ligate together and produce products that can be quantified by real-time PCR. A significant advance was the development of Hi-C using next-generation sequencing as a high-throughput way of examining the whole genome [82, 83]. Complementing this, in 2014, Hughes and colleagues [84] developed Capture C allowing analysis of hundreds of target regions at high resolution. In Capture C, a 3C library is generated by sonicating DNA into small fragments and the ligation adaptors. An oligonucleotide capture is then used by binding a region with a known sequence, usually an enhancer or promoter of interest. This is followed by library amplification, and finally, paired-end sequencing to show which enhancers and promoters physically interact with certain loci, indicating a *cis*-interaction. For diseases like AS where large numbers of variants need to be analysed, chromatin conformation capture is potentially useful in providing evidence for the potential regulatory mechanism of SNPs identified through GWAS.

Chromatin conformation capture technology has already proven instrumental in understanding regulatory effects in other diseases. Smemo *et al.* [85] used this approach in studying obesity, to undercover an interaction occurring between associated variants in an intron and a distant enhancer. Previous GWAS data had indicated that variants within introns of the *FTO* gene were associated with obesity [86–88]. *FTO* was then found to encode an enzyme involved in metabolism and regulation of body weight [89, 90]. Initial efforts focused on whether these variants modulated *FTO* gene expression. Smemo *et al.* [85] then performed a 3C analysis on mice and found that the intronic region of *FTO* containing these variants was interacting with the *Irx3* gene, encoding a homeodomain transcription factor, located over 500 kb away. In human brain samples tested, these obesity-related variants showed an association with expression levels of *IRX3*, but surprisingly not with *FTO* as previously predicted. Continued investigation into *Irx3* showed that a 30% reduction in body weight occurred in mice with an *Irx3* knockout, further validating this interaction. The use of 3C allowed for the discovery of a new role for *IRX3*, as it had never before been associated with obesity. This finding demonstrated the power of chromatin conformation capture to uncover unique regulatory interactions occurring at great distances across the genome, and this technology is likely to be instrumental in defining regulatory variants in other complex disorders, such as AS.

### Mapping gene expression as a quantitative trait

Expression quantitate trait loci (eQTL) mapping has been another valuable tool in understanding the function of non-coding variants by establishing genetic association for a given variant with differences in gene expression. eQTL have been found to be often highly context specific, dependent on cell and tissue type [91–93] as well as activation state [94, 95] and disease context [96]. The Genotype-Tissue Expression (GTEx) study provides an extremely powerful resource for exploring tissue-specific effects, including eQTL for 53 different tissues [97, 98]. AS is an example of a disease in which the most relevant cell type to disease pathogenesis remains unresolved, as well as the specific disease-relevant conditions to evaluate how variants may be affecting gene expression, although there are now diverse strands of evidence prioritizing specific cell types (discussed in the following section). Further work evaluating eQTL using cells and tissue from patients with active disease could provide the most accurate representation of the correlation between genotype and gene expression.

## Applying -omic approaches in context: biology of AS

The tools described above can be useful in helping us understand GWAS variants, but the value of the data produced relies heavily on how relevant the cell and tissue types from which the data were collected are for disease. Major cell types involved in autoimmune disease, and potentially important in AS, are T cells, natural killer (NK) cells, neutrophils, dendritic cells, monocytes and B cells [99]. Evidence for the involvement for specific circulating immune cell types in AS, which are accessible to investigators for functional genomic studies, is outlined below recognizing that future work on synovial tissue as well as cartilage and connective tissue around the facet joints of the spine and sacroiliac joints will be essential and aided by development of minimally invasive methods for sampling.

One theory of AS pathogenesis, known as the arthritogenic peptide hypothesis, relies on the involvement of CD8+ T cells [99, 100]. This hypothesis suggests that the self-peptides displayed by HLA-B27 bear a resemblance to peptides produced by foreign microbes and become the target of autoreactive CD8+ T cells. These cells then initiate an immune response and inflammation ensues. Moreover, it was found that CD4+ T cells express KIR3DL2, which is a receptor that recognizes homodimers of HLA-B27 but does not recognize heterodimers of HLA-B27 [101]. Interestingly, it was found that AS patients who are HLA-B27 positive have an increased number of cells expressing KIR3DL2. The binding of KIR3DL2 to HLA-B27 homodimers could trigger an immune response and lead to inflammation, and it is also suggested that binding triggers Th17 responses [31]. These theories suggest a connection between T cells and HLA-B27, but there are other factors involved in inflammation relevant to AS.

There is much evidence on the role of IL-23 in AS pathogenesis [9, 17]. To produce an inflammatory response, IL-23 binds to its receptor, IL-23 R, which then induces IL-17. IL-17 activation leads to the production of IL-6 and IL-8, which are major contributors to inflammation. T helper 17 (Th17) cells are the main contributors of IL-17, but there is evidence that it is also secreted by NK cells, mast cells, CD4+ T cells and neutrophils [102, 103]. Th17 cells are promising as a key cell type involved in AS pathogenesis, as they are involved in recruiting neutrophils and monocytes to a site of inflammation and encourage the development of osteoclasts, which absorb bone tissue, potentially one of the triggers for bone growth to occur at a later stage [104]. More evidence supporting the role of Th17 cells is that high levels of Th17 cells have been found in the peripheral blood of AS patients and that anti-TNF therapy, which is the most effect current treatment available for AS, reduces the number of Th17 cells in the blood [105].

Functional genomics studies have also provided evidence for the involvement of T cells in AS. Farh *et al.* [106] conducted a study to investigate fine mapped GWAS SNPs across immune diseases. To understand which cell types were contributing to disease, they performed a comparative analysis between SNP location and chromatin maps showing *cis*-regulatory elements across different cell types and found enrichment for those in Th17, Th0, Th1 cells and monocytes in AS. Monocytes in spondyloarthritis patients exhibit an upregulation of genes involved in essential inflammation pathways. One study used label-free quantitative expression profiling to examine protein expression [107]. The authors noted upregulation of proteins involved in leukocyte recruitment, and important signalling pathways, such as vascular endothelial growth factor, Janus kinases/Signal Transducer and Activator of Transcription proteins and toll-like receptor in monocytes from AS patients. Moreover, there was upregulation of genes in the ubiquitin proteasome pathway in AS monocytes [107]. This pathway is involved in the formation of peptides to be presented by HLA Class I proteins, like HLA-B27.

In terms of other circulating immune cells, one study looked at the involvement of regulatory B cells in AS and found that there was a defect in these B-cell populations in AS [108]. B cells can suppress T cells by secreting IL-10 [109]. The authors found that IL-10 secreting B cells were reduced in AS and found that controls were better able to suppress memory CD8+ T cells and naïve T cells [108]. The same study also found that B-cell population numbers were increased in AS, suggesting that they may play a significant role in disease. As mentioned above, IL-17 is a key regulator of inflammation, and there is evidence that B cells are receptive to it and are known to accumulate at the site of inflammation [102]. This could be further evidence implicating B cells in AS pathogenesis. It has been suggested that there are specific receptors found on NK cells, involved in the innate immune response and inflammation, that are able to interact with homodimers of HLA-B27 and contribute to AS [101]. Dendritic cells are important in antigen presentation and also secrete IL-23, which is thought to be a major signalling pathway in AS [110]. Further knowledge is needed relating to which cells are the most involved in the inflammation and bone growth occurring in AS.

Given this current heterogeneity in terms of the key immune cell types in AS, single-cell genomics is likely to significantly impact on our understanding of the functional genomics of disease [111]. While single-cell transcriptomics has been widely adopted, opportunities for epigenomic profiling are increasing. For example, Buenrostro *et al.* [112] used a microfluidic approach to isolate single cells from eight different cell types and then performed ATAC-seq. They believed that *trans*-factors would cause variability in chromatin accessibility between individual cells and found that this was related to specific transcription factor binding, for example, *GATA1* and *GATA2* in K562 cells. They also found that this cell-to-cell variation is related to cell type. *NFKB*, one of the major inflammatory mediators, seemed to increase chromatin state variability between cells in one cell type but not in others. Epigenetics was further examined by investigators looking at protein binding and control of regulatory elements at a single-cell level [113]. They studied H3K4me2 in embryonic stem (ES) cells and found that enhancer and promoter occupancy varied between cells, and they were able to categorize cells from the ES population based on epigenetic profile into three groups with different protein binding. These studies suggest that looking at genomics from a single-cell point of view can provide us with a new depth of information. In the context of AS, this technology could be used to examine expression and epigenetic profiles from different cell types or tissues to help resolve relevant cell types and context-specific interactions.

## Assigning causal relationships for disease-associated variants

Our current tools used for evaluating GWAS hits can help to identify the genes and genomic regions that variants are interacting with and how they may influence gene expression, but they do not necessarily provide causal evidence for mechanism. Advances in genome editing based on clustered regularly interspaced short palindromic repeats (CRISPR) are a powerful approach to address this challenge. CRISPR are short segments of prokaryotic DNA that, when combined with CRISPR associated protein 9 (Cas9) and specific guide RNAs (gRNAs), have the ability to selectively bind to regions of DNA and create a double-stranded break [114–116]. Once this break has been created, the cell will repair the damage via non-homologous end joining (hence disrupting the normal sequence and sucessfully used to knockout genes), or homology-directed repair to specifically introduce a new sequence of interest [114–116]. This method has been rapidly gaining momentum because of its ease of use and efficiency, and is replacing zinc finger nucleases and transcription activator-like effector nucleases as a means of genome editing. Using data generated through chromosome conformation capture, ATAC-Seq and eQTL mapping can help researchers generate an informed hypothesis that can then be tested by producing these mutations using CRISPR/Cas9, making it a highly valuable tool. Fine-mapped GWAS SNPs of interest and regulatory elements could be tested by editing within an appropriate cell type or animal model to experimentally validate its mechanistic effect.

This approach has already been used with success by Soldner *et al.* [117] in 2016 in Parkinson’s disease. The authors were able to uncover a risk variant in a non-coding enhancer element that regulates *SNCA* expression by creating multiple SNPs of interest using CRISPR/Cas9, and they then measured expression. It was found that a SNP, rs356168, lies within an enhancer outside of the *SNCA* gene, which is associated with Parkinson’s disease. The A allele of this SNP is protective and enables binding of a transcription factor to control the expression of SNCA. The G allele, however, prevents this transcription factor from binding and causes upregulation of *SNCA*, leading to disease. This causative SNP was in LD with others, and by using CRISPR, the investigators were able to determine which SNP was disease relevant. Another study using CRISPR to examine putative causal SNPs for autoimmune diseases also proved successful when investigators related the risk variant, rs6927172, to the expression of *TNFAIP3* and *IL-20RA*, which had not been previously associated with immune disease [118]. Unfortunately, while highly informative, generating and testing variants in this way is currently low throughput.

Genome-wide CRISPR/Cas9 screens are a higher-throughput approach. CRISPR screens involve knocking out many genes or creating mutations in one experiment. They involve designing a library of gRNAs and then transducing cells at a low multiplicity of infection to ensure that one cell will only uptake one gRNA [119]. The cells are then selected using a selection marker or treatment, and sequenced to determine which gRNAs led to the desired phenotype. Such screens can also be applied to characterize the non-coding genome [120]. One example involved characterising non-coding regions that may be influencing gene expression leading to melanoma drug resistance [121]. The investigators designed gRNAs in the regions surrounding three genes already associated with vemurafenib drug resistance in melanoma through a CRISPR/Cas9 knockout screen [122]. Melanoma cells containing a *BRAF* mutation, which is inhibited by vemurafenib, were transduced with these gRNAs and then treated with vemurafenib for selection. The cells resistant to this treatment were mainly enriched for gRNAs surrounding the *CUL3* gene and decreased expression of this gene, leading to the assumption that these are key regulatory areas in gene expression and drug resistance.

Researchers have also started moving beyond conventional CRISPR and modifying Cas9 to do more than just cleave DNA. Simeonov *et al.* [123] implemented a CRISPR activation (CRISPRa) method to uncover novel enhancers that may only be active under certain conditions. Instead of generating a double-stranded break, the Cas9 is fused to VP64, which is used for transcriptional activation. CRISPRa in this experiment identified novel elements within the *IL2RA* super-enhancer responsible for inducible expression of *IL2RA*. The ability to study inducible enhancers without needing to know the context in which they are active can be used in the study of immune disease in which enhancers are often only active when stimulated with molecules such as lipopolysaccharide. In addition to activation, genes can also be repressed through the use of modified Cas9 by methylation or deacetylation of histones at a specific locus of interest [124, 125].

## Conclusions

GWAS has been a powerful tool in uncovering genetic risk variants for complex disorders, however refining GWAS variants remains a significant challenge. In this review, we have outlined the techniques being implemented in post-GWAS studies to close the gap between finding a disease-associated variant and identifying the causal variant(s), with particular reference to AS. Fine mapping is an essential step and provides a statistical approximation of those variants most likely to be disease causing. With most of the GWAS variants being located in non-coding regions, understanding the chromatin landscape is important. Techniques such as chromatin conformation capture can help us understand physical interactions occurring in the chromatin, while other approaches such as ATAC-seq establish where regions of open chromatin are located. eQTL data can further suggest regulatory elements by providing a link between the expression of a specific gene and a genetic variant. This information can then be combined to generate hypotheses about the mechanism of action of variants, which can be experimentally validated in a biological system using CRISPR/Cas9 technology. However, challenges remain in understanding context specificity of variant interactions and knowing what the relevant cell types and conditions are for biological assays to best mimic disease. Using this approach to functionally validate GWAS variants is key to understanding the underlying genetic aetiology of AS and to using genetic data to identify and prioritize drug targets.

**Key Points**

- AS is a highly heritable multifactorial trait with multiple genomic loci implicated by GWAS.
- Functional genomic approaches in disease-relevant cell and tissue types are needed to generate hypotheses regarding the likely modulated gene(s) and mechanism of action.
- Genome editing approaches such as using CRISPR/Cas9 provide new ways of testing hypotheses by examining the biological effects of GWAS variants in model systems to establish causal relationships.

## Funding

This work was supported by Arthritis Research UK (grant number 20773 to J.C.K.); Wellcome Trust Investigator Award (grant number 204969/Z/16/Z to J.C.K.), Wellcome Trust Grant (grant number 090532/Z/09/Z to core facilities Wellcome Centre for Human Genetics) and the NIHR Oxford Biomedical Research Centre.

## Notes

**Julie Osgood** is a doctoral student at the University of Oxford conducting research into the functional genomics of ankylosing spondylitis.

## Notes

**Julian Knight** is Professor of Genomic Medicine at the University of Oxford. His laboratory studies the impact of genetic variation on the innate immune response.
